# Identification of outliers and positive deviants for healthcare improvement: looking for high performers in hypoglycemia safety in patients with diabetes

**DOI:** 10.1186/s12913-017-2692-3

**Published:** 2017-11-16

**Authors:** Brigid Wilson, Chin-Lin Tseng, Orysya Soroka, Leonard M. Pogach, David C. Aron

**Affiliations:** 1 IIRECC – EUL 5M677, Louis Stokes Cleveland Department of Veterans Affairs Medical Center, 10701 East Blvd., Cleveland, OH 44106 USA; 2 Department of Veterans Affairs-New Jersey Healthcare System, East Orange, NJ USA; 3 Department of Veterans Affairs - Office of Specialty Care Services, Washington DC, USA; 4 Case Western Reserve University School of Medicine, Cleveland, OH USA

**Keywords:** Diabetes, Overtreatment, Best practices, Positive deviance

## Abstract

**Background:**

The study objectives were to determine: (1) how statistical outliers exhibiting low rates of diabetes overtreatment performed on a reciprocal measure – rates of diabetes undertreatment; and (2) the impact of different criteria on high performing outlier status.

**Methods:**

The design was serial cross-sectional, using yearly Veterans Health Administration (VHA) administrative data (2009–2013). Our primary outcome measure was facility rate of HbA1c overtreatment of diabetes in patients at risk for hypoglycemia. Outlier status was assessed by using two approaches: calculating a facility outlier value within year, comparator group, and A1c threshold while incorporating at risk population sizes; and examining standardized model residuals across year and A1c threshold. Facilities with outlier values in the lowest decile for all years of data using more than one threshold and comparator or with time-averaged model residuals in the lowest decile for all A1c thresholds were considered high performing outliers.

**Results:**

Using outlier values, three of the 27 high performers from 2009 were also identified in 2010–2013 and considered outliers. There was only modest overlap between facilities identified as top performers based on three thresholds: A1c < 6%, A1c < 6.5%, and A1c < 7%. There was little effect of facility complexity or regional Veterans Integrated Service Networks (VISNs) on outlier identification. Consistent high performing facilities for overtreatment had higher rates of undertreatment (A1c > 9%) than VA average in the population of patients at high risk for hypoglycemia.

**Conclusions:**

Statistical identification of positive deviants for diabetes overtreatment was dependent upon the specific measures and approaches used. Moreover, because two facilities may arrive at the same results via very different pathways, it is important to consider that a “best” practice may actually reflect a separate “worst” practice.

## Background

Learning from high performing health care systems constitutes an important strategy for organizational improvement [1–4]. Among the methods used to identify such systems is the identification of statistical outliers based on specific performance measures [5–7]. Identification of outliers has also constituted the first step in the identification of “positive deviants,” a strategy that has gained popularity in healthcare improvement [8–14]. This approach depends upon the choice of a robust measure that accurately represents performance. However, criteria for outlier status remain uncertain [5–7]. Moreover, in complex disease management individual measures reflect only one aspect of performance. For example, undertreatment and overtreatment are each associated with adverse outcomes. A focus on undertreatment of a particular condition to reduce one set of adverse outcomes can result in overtreatment of some patients resulting in increase in another set of adverse outcomes [15, 16]. Measurement of both undertreatment and overtreatment would better reflect organizational performance than measurement of only one. This is particularly relevant to diabetes.

The National Committee for Quality Assurance (NCQA), National Quality Forum (NQF) and others have developed a variety of performance measures related to diabetes [17]. Central to such assessment has been measures of glycemic control, with a focus on rates of under-treatment [18, 19]. A1c targets are typically individualized at different levels within the range of <7% to <9%. However, undertreatment has typically been assessed relative to the high end of that range, i.e., by measures such as the percentage of patients with diabetes with A1c > 9% and there have been efforts to address this undertreatment for many years. More recently, greater attention has been paid (in terms of performance measurement) to undertreatment at the low end of the range, i.e., the percentage of patients with A1c > 7%. The American Diabetes Association recommended A1c <7% for all patients 19–74 years of age. In May 2006 the NCQA included measures of optimal glycemic control (A1C <7%) for public reporting in 2008 [17]. Consequently, the potential for overtreatment became more evident [20–22]. This may occur by setting targets for glucose control that are inappropriately low based on patients’ life expectancies and/or comorbid conditions, resulting in risk for serious hypoglycemia [23–27]. In fact, this issue became the focus in 2014–5 of national initiatives including the Choosing Wisely initiative which recommends “moderate control” of A1c in most older adults, a recommendation from the American Geriatrics Association [28]. In addition, the FDA in collaboration with NIDDK, CDC, and VA included hypoglycemia safety as a major component of its 2014 Action Plan on Adverse Drug Events. Consequently, VA initiated a major effort to reduce overtreatment in 2015, the Choosing Wisely/Hypoglycemia Safety Initiative [29]. Therefore, we focused on overtreatment as an issue of patient safety. Patient safety is an area in which positive deviance has been applied and where the identification of high performing outliers is a critical first step [30]. The primary objective of our study was to determine how statistical outliers exhibiting low rates of diabetes overtreatment performed on a reciprocal measure – rates of diabetes undertreatment. Also, since different measure thresholds, different comparators, and consistency of performance over time may affect which facilities are identified as outliers, a secondary objective was to determine the extent to which high performing outlier status for diabetes overtreatment is impacted by different criteria.

## Methods

### Study design

This was a serial cross-sectional study design, using yearly Veterans Health Administration (VHA) administrative data from fiscal year (FY) 2009–2013. This study was approved by the Department of Veteran Affairs (VA)–Louis Stokes Cleveland VA Medical Center and New Jersey Health Care System Institutional Review Boards. There was waiver of informed consent.

### Study population – healthcare system

This study was carried out using data from a very large healthcare system - the Veterans Health Administration (VHA). VHA provides comprehensive healthcare to eligible veterans of the Armed Services in >100 hospitals and their related clinics. In the years of the study, it was organized into 21 regional networks (Veterans Integrated Service Networks or VISNs), each consisting of 3–10 facilities. Facilities vary by the level of complexity depending upon size, scope of clinical activities, and other site characteristics.

### Study population – patients (at risk group)

Patients who met our previously proposed criteria for a population with risk factors for hypoglycemia (hence overtreatment) were included in the study [22]. Specifically, this population included diabetic patients taking a diabetes drug known to have a relatively high frequency of hypoglycemia (insulin and/or sulfonylurea agents) plus having at least one of the following additional criteria: age 75 years or older, chronic kidney disease (defined as last serum creatinine measurement in a year greater than 2.0 mg/dL (to convert to micromoles per liter, multiply by 88.4), or an *ICD-9-CM* diagnosis of cognitive impairment or dementia in ambulatory care. Diabetes mellitus status for a given year was defined based on 2 or more occurrences of *International Classification Of Diseases, Ninth Revision, Clinical Modification* (*ICD-9-CM*) codes for diabetes mellitus (250.xx,) associated with clinical face-to-face outpatient care on separate calendar days in prior 2 years, or oral diabetes mellitus–specific medication prescription (insulin, sulfonylurea, biguanide, α-glucosidase inhibitor, meglitinide, or thiazolidinedione) in prior year. Among these patients, we retained only those that had at least one hemoglobin A1c (HbA1c) value documented in any fiscal year from FY2009-FY2013 to be the final study population (at risk group) and denominator for calculation of rates. In addition, we separately analyzed all other patients with diabetes (not at high risk group). Data sources included the VHA National Patient Clinical Data Set (Austin, Texas; to obtain *ICD-9-CM* and diagnostic codes) and the Decision Support System (to obtain laboratory data and medication information). Because veterans may obtain care from more than one facility, we determined a patient’s parent facility based on where they received most of their ambulatory care.

### Outcome measure – over- and under-treatment rates

Our primary outcome measure was rate of overtreatment of diabetes at a facility level in patients at high risk for hypoglycemia based on the criteria above, i.e., the proportion of patients with A1c < 6.5% [22]. We defined overtreatment in these patients based on their last HbA1c value in a year, consistent with industry standards. However, because dichotomous measures of A1c can be sensitive to small changes in average A1c, we also examined thresholds of A1c < 6% and A1c < 7% [31]. The former represents extremely intensive glycemic control while the latter is still a commonly used quality measure applicable to patients age < 65 years of age. A1c < 7% is also closer to the Choosing Wisely recommendation of the American Geriatrics Society [28].

Since overtreatment may be an unintended consequence of focus on undertreatment, we also examined rates of undertreatment as our secondary outcome measure, i.e., the proportion of patients with an A1C > 9% [32]. Undertreatment rates were calculated in two populations: (i) the population at risk for hypoglycemia; and (ii) all patients with diabetes not in the at risk group.

### Outcome measure – outlier status

Outlier status was assessed using two approaches: a facility outlier value measure weighted by at-risk population and standardized within year and comparator group; and model residuals. (For comparison, we also assess a facility outlier measure unweighted by the at-risk population.) For each year, A1C threshold, and a comparator, a population overtreatment rate *p* was calculated and the facility outlier value was calculated based on the difference between the observed number of overtreated patients, *x,* and the expected number under the normal approximation given the number of patients at risk, *n*, and the population overtreatment rate *p*: (*x* – *np*)/√(*np*(1-*p*)). One facility was excluded from this analysis based on small sample size (an at-risk population for which either *np* or *np(1-p)* was <5).

We utilized three comparators: (1) all VA hospitals; (2) hospitals within the same VISN; and (3) hospitals within the same complexity level. Facilities with outlier values in the lowest decile for all years of data based on more than on comparator-A1c threshold were considered high performing outliers and analyzed further.

We also utilized standardized residuals derived from linear mixed effects models controlling for complexity and VISN to determine outliers. For each year and A1c threshold, we considered a facility *j* in VISN *i* in the following model in which random intercepts were assumed for each VISN:

$$ {\mathit{\mathsf{Overtreatmentrate}}}_{\mathit{\mathsf{i}\mathsf{j}}}={\beta}_{\mathsf{0}}+{\alpha}_{\mathsf{0}\mathit{\mathsf{i}}}+{\beta}_{\mathsf{1}}\mathit{\mathsf{Complexity}}+{\varepsilon}_{\mathit{\mathsf{i}\mathsf{j}}} $$

By considering model residuals (*ε*_*ij*_) and standardizing them within year and threshold, we assessed the relative performance of facilities across years while controlling for complexity, VISN, and both the mean and variability of the VA-wide overtreatment levels in a given year. A negative residual for a facility indicates a lower than predicted rate, and a positive residual indicates a greater than predicted rate. To determine if a facility is a high performing outlier, we averaged the model residuals across time from overtreatment rates for each of the three thresholds. Facilities in the lowest decile of residuals from all three thresholds were considered to be high performing outliers and were analyzed further.

### Independent variables

For each facility, we obtained the information of its designated VHA regional service area, known as Veteran Integrated Service Networks (VISN). We also employed a measure of VHA facility complexity level from 2011. VHA classifies facilities into five complexity levels (1a, 1b, 1c, 2, and 3) based on their size, scope of clinical activities, e.g., range of specialties providing care, and other site characteristics; level 1a is most complex while level 3 is least complex. There are 18–32 facilities per complexity level.

### Statistical analyses

Overtreatment rates and at-risk populations for each of the 135 facilities were calculated for each year - FY 2009 through 2013. We utilized three A1c thresholds (6, 6.5, and 7%). Similarly, we calculated undertreatment rates in the same population using an A1c > 9% as threshold. We then assessed the VA-wide trends in these rates, calculating Spearman rank correlations of overtreatment and undertreatment rates with time.

To determine performance outliers among facilities, we first used a non-modeling approach comparing each facility rate to one (of three) comparators to obtain a facility outlier value for each year. To assess consistency of performance over time, we calculated correlation coefficients (Pearson Product moment).

Since facility-level rates may be different due to factors not related to facility efforts (hence, performance) in glycemic control, such factors may need to be considered in comparing facility performance; health care facilities differ in their levels of services provided (complexity level). We used facility complexity (see below) as a proxy for specialty care resources. In addition, VHA is organized into regional networks (VISN) where network level initiatives might result in geographic differences in performance. Thus, our second approach used linear mixed-effects models to adjust for the effects that were previously considered as comparators (facility complexity, VISN-specific trends, and VA-wide variability) simultaneously and examine the standardized residuals from such models as outlier metrics [33, 34].

For each year and A1c threshold, a linear mixed-effects model containing facility complexity as a fixed effect and a random intercept for each VISN was fit to the data of facility overtreatment rates. The distributions of residuals and their independence from predicted values were examined for all estimated models to check model assumptions. The effect of complexity was assessed in each estimated model. We chose to estimate separate models for each year so we would not need to parameterize the correlation structure for the repeated measures within facility or changes in variability across the VA over time. The use of standardized residuals allowed us to compare facilities across years and thresholds to identify those that consistently perform better or worse than expected/predicted.

All analyses were performed using SAS 9.2 and R 3.1.1 statistical software.

## Results

### Identification of outliers with low rates of overtreatment and changes over time

**Fig. 1:**
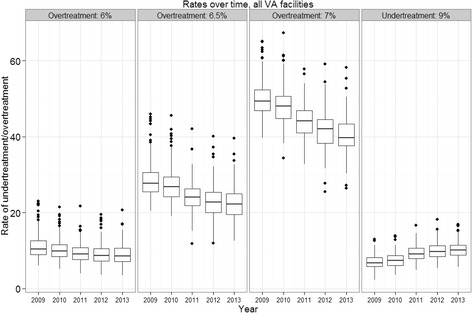
Overtreatment and undertreatment rates measured at facilities by year and threshold. For all three overtreatment thresholds, a decrease over time was observed. The rates of overtreatment increase with the overtreatment threshold as more patients are captured. The undertreatment rates are of similar magnitude to the overtreatment rates using 6% as the threshold and are increasing over time. Outliers are more present at high rates than at low rates

**Fig. 2:**
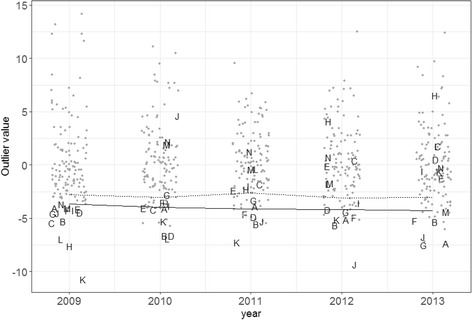
Overtreatment outlier values over time by 2009 deciles using 6.5% threshold and all VA facilities as comparator. Facilities were classified into deciles by their 2009 performance using outlier values into and then tracked using the same threshold and comparator across time to illustrate the correlations and consistency of performance over time. The solid line indicates the limit of the top decile year by year. The dotted line indicates the limit of the top two deciles. Facilities in the top decile in 2009 are labeled with letters. Over time, the performance of these facilities spread across the entire distribution

Overtreatment rates in general fell over time (Fig. 1). Facilities in the top performing decile (based on their 2009 performance using 6.5% threshold and all VA facilities as comparator) and their performance over time are shown in Fig. 2. Facility performance varied over time. Although half (7/14) of the high performers were in the top performing decile in at least 2 of the subsequent 4 years, the range of the performance increased over time; the top decile remained largely separate in 2010 and less so with each subsequent year. However, only one facility was in the top decile for all 5 years though three others were in the top two deciles. Outlier values for the same threshold in adjacent years were highly correlated and the year-to-year correlation decreased with increased separation in time (Table 1). The lowest correlations within comparator and threshold were generally seen between the 2009 and 2013 outlier values.

**Table 1:** Correlation coefficients with *p*-values for overtreatment and undertreatment rates for each year and overtreatment threshold. For overtreatment thresholds 6.5% and 7%, significant negative correlation between overtreatment rates and undertreatment rates was observed across all years; using 6% as the overtreatment threshold, correlations were negative but of lower magnitude and significance

		Separated by 1 year				Separated by 2 years			Separated by 3 years		Separated by 4 years
Comparator	A1c threshold	2009–2010	2010–2011	2011–2012	2012–2013	2009–2011	2010–2012	2011–2013	2009–2012	2010–2013	2009–2013
All VA Facilities	6%	0.67	0.72	0.82	0.8	0.54	0.61	0.58	0.4	0.45	0.29
	6.5%	0.66	0.67	0.8	0.8	0.5	0.53	0.57	0.41	0.39	0.35
	7%	0.66	0.67	0.82	0.76	0.5	0.51	0.58	0.41	0.38	0.34
Facilities within complexity level	6%	0.67	0.72	0.81	0.8	0.56	0.62	0.57	0.42	0.44	0.31
	6.5%	0.66	0.69	0.8	0.8	0.52	0.54	0.56	0.42	0.39	0.37
	7%	0.66	0.68	0.83	0.76	0.52	0.53	0.59	0.42	0.39	0.37
Facilities within VISN	6%	0.62	0.64	0.78	0.75	0.46	0.48	0.5	0.27	0.3	0.15
	6.5%	0.66	0.61	0.77	0.75	0.47	0.45	0.49	0.32	0.26	0.25
	7%	0.69	0.61	0.81	0.74	0.51	0.44	0.56	0.4	0.29	0.31

### Sensitivity of the overtreatment measure to changes in A1c threshold, facility complexity, and organizational region

**Fig. 3:**
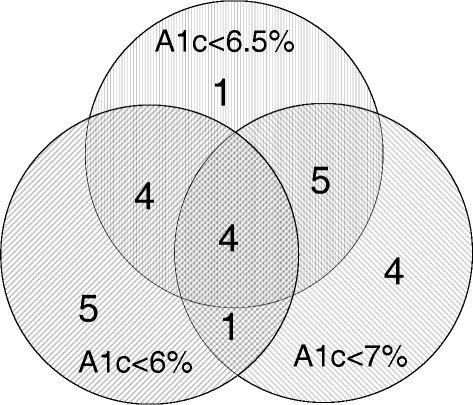
Overlap in high performing facilities across overtreatment threshold. For each threshold, the highest performing decile of facilities was identified (*n* = 14 for each threshold). Seven facilities were identified by all three overtreatment thresholds. There were no facilities identified by both 6 and 7% that were not also identified by 6.5%

There was only modest overlap between facilities identified as top performers based on three thresholds. Using 2009 outlier values based on all VA facilities and the three thresholds (A1c < 6%, A1c < 6.5%, and A1c < 7%), we looked at overlap in facilities in the highest performing decile (Fig. 3). Only 4/14 (29%) were in the top decile for all three thresholds. Similar results were observed with other years and comparators (2010–2013, VISN and complexity, data not shown). In examining the estimated effects from the overtreatment linear mixed models, facility complexity was not a significant predictor of overtreatment and inter-VISN variability consistently exceeded intra-VISN variability (Table 2).

**Table 2:** Correlations of outlier values between years for each comparator and threshold. To describe the year-to-year relationships in overtreatment performance (as measured by outlier values), correlation matrices were generated. The diagonal values within each 4-by-4 threshold/comparator combination show that outlier values in adjacent years are highly correlated and that the year-to-year correlation decreases with increased separation. The lowest correlations within each threshold and comparator are generally seen between 2009 and 2013. The correlations calculated using all VA facilities and facilities of the same complexity as the comparator are very consistent; the VISN comparator correlations are generally lower

Year	Threshold	Complexity p-value (F-test, 4 df)	Intra-VISN variability/total variability
2009	6	0.09	0.22
	6.5	0.27	0.20
	7	0.62	0.10
2010	6	0.03	0.31
	6.5	0.11	0.24
	7	0.27	0.20
2011	6	0.66	0.29
	6.5	0.49	0.28
	7	0.55	0.22
2012	6	0.90	0.27
	6.5	0.97	0.24
	7	0.93	0.23
2013	6	0.16	0.22
	6.5	0.74	0.25
	7	0.84	0.24

### Undertreatment rates in outlier facilities with low rates of overtreatment

**Fig. 4:**
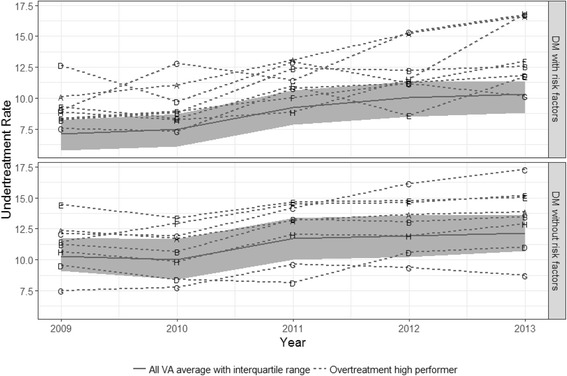
Undertreatment rates over time among facilities with lowest rates of overtreatment (dashed lines) and the average rate among all VA facilities (solid line); the shaded area represents the 95th percentile confidence interval.The upper panel shows results from the at risk population and the lower panel shows the response in the population not at high risk. Eight facilities (labeled with letters) were identified as high performers in 2009 using overtreatment metrics; but low overtreatment observed in a facility with high rates of undertreatment is of concern. Looking at the undertreatment rates over time of the flagged overtreatment facilities and comparing them to the undertreatment rates of all VA facilities, several high performers with regard to overtreatment have consistently high rates of undertreatment

Undertreatment rates as defined by A1c > 9% rose modestly over time (Fig. 4). This applied to both the population at high risk for hypoglycemia and the diabetic population not at high risk. We identified 8 consistent high performing outliers for overtreatment (*n* = 8): 7 identified using model residuals, 3 identified using outlier values, with 2 facilities identified through both approaches. These differences in outlier sets highlight the differences in the two approaches: one incorporated the size of the at-risk population and decreased variability in rates measured with larger samples while the other considered all facility rates of equal weight and validity; one required high performance in all 5 years while the other averaged performance across time.

The outliers generally had higher rates of undertreatment than the VA average in the population of patients at high risk for hypoglycemia. However, one facility with low overtreatment rates in the at risk patients was a consistent high performer in terms of undertreatment rates both in the at risk population (average rate) and not at risk populations (below average rate). The differences were less dramatic in those patients not at high risk for hypoglycemia. Correlations between overtreatment rates and undertreatment rates were calculated for each year and threshold within the at risk population. For overtreatment thresholds 6.5% and 7%, significant negative correlations between overtreatment rates and undertreatment rates were observed across all years (ρ ranging from −0.5 to −0.3); using 6% as the overtreatment threshold, correlations were negative but of lower magnitude and not significant (ρ ranging from −0.2 to −0.1).

## Discussion

Our results demonstrate two important findings and add to literature on the impact of using different measures or different statistical approaches on hospital rankings and outlier status [10, 22, 35–37]. First, our results indicate the importance of having a balancing measure. Without such a measure, one can be misled into thinking that a high-performing hospital, e.g., one with very good results with respect to overtreatment might be a good performer with respect to undertreatment. While overtreatment in general fell over time and undertreatment rose, we found that those high performing facilities in overtreatment tended to exhibit a trend of increasing undertreatment over time that often exceeded the VA-wide average. Such facilities should not be considered positive deviants. A low rate of overtreatment may be observed under both desirable and undesirable conditions. Ideally, low rates of overtreatment occur because of specific attention to patients at risk for hypoglycemia. Less ideally, a low rate of overtreatment may occur as an artifact of widespread under-treatment and a facility-wide tendency towards higher A1c levels. In fact, the reverse has been observed where overtreatment may be an unintended consequence of focus on undertreatment [15, 16].

Second, our findings indicate that the statistical identification of high performing outliers is very sensitive to the specific criteria chosen, i.e., the statistical approaches to identifying positive deviants may not be very robust. For example, the choice of three different A1c levels to define overtreatment resulted in three series of high performing outliers with only modest overlap. This variation has implications for the validity of conclusions drawn from league tables, particularly those based on a single measure, and highlights the need to understand the clinical differences of thresholds when interpreting differences in quality results seen across thresholds [7, 38]. Moreover, our findings indicate the importance of identifying consistent high performance. A positive deviant in 1 year may not merit that designation in other years. This issue is particularly true for dichotomous (threshold) measures applied to continuous outcomes where small changes in statistical distribution may have a large effect on the measure. Interestingly, there was little impact of facility complexity or VISN on the findings; limiting comparators to like facilities was not necessary in this particular circumstance. It may be that the lack of effects of facility characteristics reflects the fact that most patients with diabetes are managed by primary care providers even when a facility has a diabetes specialist. Primary care services are available at all facilities regardless of the overall scope of services provided by the facility.

Although we used dichotomous thresholds in our analyses, it is important to recognize that quality measures vary depending upon the specific issue. For example, wrong site surgery is a “never event” and a lower rate is always better. We chose our at-risk population to make A1c < 6% essentially a never event (with A1c, this is more controversial). In these circumstances, it is important prove a balancing measure. But there are patients in this group for whom an A1c < 7% might be appropriate. The U/J-shaped curves for mortality and body mass index and blood pressure also suggest that lower is not always better [39, 40].

Finally, our results have implications for the increasingly popular “positive deviance” approach to improvement [41, 42]. The “positive deviance approach” to social/behavior change began in the area of childhood nutrition when it was noted that within communities with high levels of childhood malnutrition some families had well-nourished children [43, 44]. Families referred to as positive deviants evidenced uncommon but successful behaviors or strategies that enabled them to find better solutions to a problem than their peers, despite facing similar challenges and having no apparent extra resources or knowledge. In the original work on positive deviance, performance was assessed by simple observation rather than statistical analysis; the difference between malnourished and well-nourished children was obvious [43]. Reliance on simple observation may not be a feasible strategy for identifying organizational positive deviance. Moreover, when applied in healthcare, there has been variation in the criteria for deviance (both magnitude and comparator), and the level (individual, team, unit, or organization) at which it is applied [8, 10, 22, 43]. In the original work on positive deviance in public health, the comparators were required to be those with similar access to resources. In contrast, Krumholz et al. selected hospitals that were diverse in areas such as the volume of patients with acute myocardial infarction, teaching status, and socioeconomic status of patients [10]. Later studies examined what the positive deviants were doing that was different from other intensive care units and those actions were shared with others [9, 45]. Many hospitals have since adopted those practices with resulting improvement in outcomes. However, despite their good results, several questions are raised about extrapolating that approach to other issues [46, 47]. Although there are numerous methods for outlier detection and differences in both criteria and comparator, our study suggests that considerable thought needs to be given to this issue at the outset, before attempts are made to identify performance outliers.

### Limitations

This study has several limitations. First, this study involved assessment of the management of a single issue, which is multidimensional and involves patients, providers, and organizations and all of their interactions. Nevertheless, the condition chosen is a common one and is currently the subject of a national initiative because of its importance. Second, there are many ways to identify outliers statistically. However, by necessity, we limited our statistical analyses so that we cannot infer that other types of analyses would exhibit the same findings. Nevertheless, we used methods that are commonly employed and thus familiar to those for whom assessing performance is important. The study was limited to a single healthcare system, albeit a very large one. It may be that factors unique to this have an impact on the findings. Finally, we focused on the first part of the “best practices” approaches – the identification of deviants or high performing sites and not on the best practices themselves, i.e., what the practices actually were. Similarly, we did not address the issue of implementation of the practices elsewhere, which is a critical part of the positive deviance approach. Notwithstanding these limitations, we believe we have illustrated some of the issues involved in using the positive deviance or best practices approaches.

## Conclusions

In summary, we have found that in the case of overtreatment of diabetes in the Veterans Healthcare System, statistical identification of high performing facilities was dependent upon the specific measures used. This variation has implications for the validity of conclusions drawn from league tables based upon single measures. Moreover, these results combined with the literature extant suggest that the choice of comparator is dependent upon the nature of the practice. Finally, because two facilities may arrive at the same results via very different pathways, it is important to consider that a “best” practice may actually reflect a separate “worst” practice.

## Abbreviations

FY: Fiscal year
HbA1c: Hemoglobin A1c
ICD-9-CM: International Classification Of Diseases, Ninth Revision, Clinical Modification
NCQA: National Committee for Quality Assurance
NQF: National Quality Forum
VA: Department of Veteran Affairs
VHA: Veterans Health Administration
VISN: Veterans Integrated Service Network
